# Direct targeted therapy for MLL‐fusion‐driven high‐risk acute leukaemias

**DOI:** 10.1002/ctm2.933

**Published:** 2022-06-22

**Authors:** Sandra Cantilena, Luca Gasparoli, Deepali Pal, Olaf Heidenreich, Jan‐Henning Klusmann, Joost H. A. Martens, Alexandre Faille, Alan J. Warren, Mawar Karsa, Ruby Pandher, Klaartje Somers, Owen Williams, Jasper de Boer

**Affiliations:** ^1^ Cancer Section, Development Biology and Cancer Programme UCL GOS Institute of Child Health London UK; ^2^ Newcastle Cancer Centre at the Northern Institute for Cancer Research Newcastle University Newcastle upon Tyne UK; ^3^ Department of Pediatrics I Martin‐Luther‐University Halle‐Wittenberg Halle Germany; ^4^ Department of Molecular Biology, Faculty of Science, Radboud Institute for Molecular Life Sciences Radboud University Nijmegen The Netherlands; ^5^ Cambridge Institute for Medical Research Cambridge UK; ^6^ Department of Haematology University of Cambridge Cambridge UK; ^7^ Wellcome Trust–Medical Research Council Stem Cell Institute University of Cambridge Cambridge UK; ^8^ Children's Cancer Institute, Lowy Cancer Research Institute University of New South Wales Randwick New South Wales Australia; ^9^ School of Women's and Children's Health University of New South Wales Randwick New South Wales Australia; ^10^ Present address: Victorian Comprehensive Cancer Centre Alliance Melbourne Australia

**Keywords:** leukaemia, MLL‐fusion, mouse models, precision medicine, targeted therapy

## Abstract

**Background:**

Improving the poor prognosis of infant leukaemias remains an unmet clinical need. This disease is a prototypical fusion oncoprotein‐driven paediatric cancer, with *MLL* (*KMT2A*)‐fusions present in most cases. Direct targeting of these driving oncoproteins represents a unique therapeutic opportunity. This rationale led us to initiate a drug screening with the aim of discovering drugs that can block MLL‐fusion oncoproteins.

**Methods:**

A screen for inhibition of MLL‐fusion proteins was developed that overcomes the traditional limitations of targeting transcription factors. This luciferase reporter‐based screen, together with a secondary western blot screen, was used to prioritize compounds. We characterized the lead compound, disulfiram (DSF), based on its efficient ablation of MLL‐fusion proteins. The consequences of drug‐induced MLL‐fusion inhibition were confirmed by cell proliferation, colony formation, apoptosis assays, RT‐qPCR, in vivo assays, RNA‐seq and ChIP‐qPCR and ChIP‐seq analysis. All statistical tests were two‐sided.

**Results:**

Drug‐induced inhibition of MLL‐fusion proteins by DSF resulted in a specific block of colony formation in *MLL*‐rearranged cells in vitro, induced differentiation and impeded leukaemia progression in vivo. Mechanistically, DSF abrogates MLL‐fusion protein binding to DNA, resulting in epigenetic changes and down‐regulation of leukaemic programmes setup by the MLL‐fusion protein.

**Conclusion:**

DSF can directly inhibit MLL‐fusion proteins and demonstrate antitumour activity both in vitro and in vivo, providing, to our knowledge, the first evidence for a therapy that directly targets the initiating oncogenic MLL‐fusion protein.

## Graphical abstract

Direct inhibition of MLL‐fusion proteins is a potential therapeutic strategy.

MLL‐fusion depletion screen identified disulfiram for its ability to ablate MLL‐fusion protein and block‐associated leukaemic phenotype.

Disulfiram directly targets the CXXC domain that is essential for all MLL‐fusion proteins.

Disulfiram prevents the binding to the target genes of the MLL‐fusion resulting in rapid decrease in H3K27ac levels.

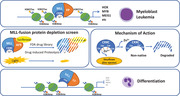

## 1 BACKGROUND

11q23 chromosomal translocations are found in both acute lymphoid leukaemias (ALLs) and acute myeloid leukaemias (AMLs). These chromosomal translocations are the basis of leukaemia in up to 80% of infant ALL and about 15%–20% of paediatric AML.^1^, ^2^, ^3^ It is a common underlying cause of leukaemia in patients who suffer from therapy‐induced AML after the treatment of an unrelated malignancy.^4^ 11q23 translocations are also sporadically detected in adult patients suffering from ALL and AML. All these leukaemic patients have in common a dismal prognosis. For instance, the 5‐year survival rate of infant ALL patients with 11q23 translocations is 15%–50%, even with the latest cutting‐edge therapies.^5^ This is significantly lower than that of infant ALL patients without 11q23 translocations which have an event‐free survival rate of 70%–90%. The unmet clinical problem presented by 11q23 translocated leukaemias was emphasized by the World Health Organization (WHO) which has classified leukaemias with 11q23 translocations as one group, with an estimated 4‐year event‐free survival of 24%–55%.^6^

The most common 11q23 translocations are balanced chromosomal translocations that fuse 5′‐sequences of the *MLL1* gene (now also known as *KMT2A*) in frame to over 80 different fusion partner genes.^7^ These MLL fusions activate a haematopoietic stem cell‐like transcriptional programme in concert with inducing an underlying gross defect in chromatin structure.^8^ *MLL* fusions and other fusion genes (e.g. *PML‐RARA*, *BCR‐ABL*) are the driving oncogenes in many leukaemias.^9^ Indeed, leukaemic cells depend on the continued expression of a driving oncogene, such that the inactivation of the oncogene product triggers a block in leukaemia progression. This was shown in acute promyelocytic leukaemia (APL), a haematological malignancy driven by the *PML–RARA* oncogene.^10^ Previously, this disease had a very poor prognosis. Currently, over 90% of APL patients are cured through the introduction of arsenic and retinoic acid to the therapy.^11^, ^12^ This revolutionary outcome was explained by the ability of these two agents to directly degrade the PML–RARA oncoprotein.^13^ Another example of fusion protein inactivation came from leukaemic patients who have a translocation between chromosomes 9 and 22, thereby creating the *BCR–ABL* oncogene. *BCR–ABL* ^+^ acute leukaemias are rarely cured with chemotherapy. However, long‐term survival is now being routinely achieved when such patients are treated with combinations of chemotherapy and BCR–ABL inhibitors (such as Imatinib and Dasatinib).^14^, ^15^ Our previous studies have shown that the inhibition of MLL‐fusions, in a conditional mouse model of MLL–ENL‐driven AML, resulted in a block in self‐renewal, increased the differentiation of the leukaemic cells resulting in the ablation of leukaemia in the mice.^16^ Furthermore, small molecular inhibitors for DOT1L, BRD4 and MENIN that target MLL‐fusion‐dependent gene pathways have shown efficacies in preclinical models of MLL leukaemia.^17^, ^18^, ^19^ The strong evidence that fusion protein inactivation can give long‐term survival in patients suffering from acute leukaemia led us to investigate if this approach could be extended to pharmacological inactivation of the MLL‐fusion proteins.

## 2 METHODS

### 2.1 DNA constructs and generation of leukaemic fusion gene cell lines

The MLL‐AF9‐fusion gene was fused to firefly luciferase using a PCR‐based cloning approach and subcloned into a pBlueScript II SK(+). The MLL‐AF9‐luciferase (MA9Luc) was subcloned into a pMSCVneo retroviral vector (pMSCVneo‐MA9Luc). The Renilla luciferase was cloned into a pMSCVpuro vector (pMSCVpuro‐Ren). The human leukaemic cell line THP‐1 was transduced with pMSCVneo‐MA9Luc, and the transduced cells were selected with neomycin. Selected cells were single cell sorted by flow cytometry, and clones were screened for the desired phenotype: firefly luciferase activity. The selected clones were then transduced with pMSCVpuro‐Ren and selected with puromycin. Selected cells were single cell sorted by flow cytometry, and cells were screened for the desired phenotype: firefly luciferase activity and renilla luciferase. This resulted in the generation of a number of indicator THP‐1 clones expressing firefly and renilla luciferases.

### 2.2 Cell lines and cell culture

Human leukaemic cell lines SHI‐1, MV4‐11, OCIAML3 and KASUMI1 were obtained from the German Collection of Microorganisms and Cell Cultures (DSMZ). The THP‐1 cell line was obtained from the American Type Culture Collection (ATCC, TIB‐202, www.atcc.org). The identity of human leukaemic cell lines was confirmed by STR profiling. All human leukaemic cell lines were routinely cultured in Roswell Park Memorial Institute (RPMI) medium (Sigma‐Aldrich), supplemented with 10% or 20% heat‐inactivated FBS, penicillin–streptomycin (100 μg/ml) and l‐glutamine (2 mM) (all Sigma‐Aldrich) with the exception of SHI‐1 cells which were cultured in Iscove's Modified Dulbecco's Medium supplemented with 20% heat‐inactivated FBS, penicillin–streptomycin (100 μg/ml) and l‐glutamine (2 mM) (all Sigma‐Aldrich) in a humidified chamber with 5% CO_2_ and 95% air at 37°C. Each cell line was subcultured every 3–4 days and plated according to supplier guidelines.

### 2.3 Drug screening and dual luciferase assays

This assay was carried out in white V‐bottom 96 well plates seeded with 1000 THP‐1 cells expressing simultaneously firefly luciferase and renilla luciferase (THP‐1‐MA9‐Luc‐Ren) per well (Non‐TC treated, Greiner Bio‐One) followed by 6‐h incubation with drugs from the Prestwick Chemical Library (1 μM) (Prestwick Chemical). Cells were washed with PBS and lysed in passive lysis buffer (1×) (Promega). Firefly luciferase buffer (Tricine 20 mM, MgSO_4_ 2.67 mM, EDTA .1 mM, DTT 33.3 mM, coenzyme A 270 μM, d‐luciferin 470 μM, ATP 530 μM, pH 7.8) was injected first into each well by the injector of the Infinite 200 PRO plate reader (Tecan). After quantifying the firefly luminescence, the renilla luciferase buffer (K_3_PO_4_ 220 mM, NaCl 1.1 M, EDTA 2.2 mM, BSA .44 g/L, NaN_3_ 1.3 mM, coelenterazine 1.43 μM) was dispensed in each well and the renilla luminescence was recorded. The ratios of firefly to renilla luminescence were calculated for each well and normalized to the control samples.

### 2.4 Isolation of human CD34^+^ cord blood cells

Human cord blood CD34^+^ cells (ZenBio Inc., Research Triangle Park, NC, USA) were purified by magnetic activated cell sorting (MACS) using the human CD34 MicroBead Kit (Miltenyi Biotec) according to manufacturer's protocol.

### 2.5 Colony forming assays

Human leukaemic cell lines were plated and cultured for 2 weeks in HSC002 (R&D system). CD34^+^ cord blood–derived cells were plated and cultured in HSC005 (R&D system) for 2 weeks. Cells were cultured for two additional days in the presence of 2‐(*P*‐iodophenyl)‐3‐(*p*‐nitrophenyl)‐5‐phenyl tetrazolium chloride (10 mg/ml). Colonies were imaged using the densitometer GS800 (Bio‐Rad) and quantified using OpenCFU software (GE Healthcare).

### 2.6 Flow cytometry analysis

Human leukaemic cells viability was assessed through To‐PRO3 staining (Invitrogen). Apoptosis was determined by staining cells using the Annexin V Apoptosis Detection Kit APC (eBioscience) in combination with propidium iodide. Both analyses were performed on an LSRII analyser (BD Bioscience). Murine peripheral blood cells were stained using allophycocyanin‐conjugated anti‐Mac1 antibody. Before staining with primary antibody, cells were resuspended in PBS, .5% bovine serum albumin and .05% sodium azide. Flow cytometry was performed using the Cyan ADP analyser (Beckmann Coulter).

### 2.7 Coculture and bioluminescence assay

ALL‐MSCs coculture system and bioluminescence assays were performed as previously described.^40^ MSCs were seeded at 1 × 10^4^ cells per well in a clear bottom 96 well plate. Firefly luciferase ALL cells were seeded (5 × 10^3^ cells/well) in 96 well plates on top of the feeder layer of MSCs and incubated for 2 weeks with different doses of disulfiram (DSF). Bioluminescence was measured using the FLUOstar Omega microplate reader (BMG) following the addition of luciferin at 30 μg/ml (Promega).

### 2.8 Western blot analysis

Cell lysates were prepared using a sample reducing buffer containing DTT (200 mM), sodium dodecyl sulphate (SDS) (2%), glycerol (10% v/v), bromophenol blue (.02% v/v), and Tris–HCl, pH 6.8 (125 mM). Samples were pelleted via centrifugation and equal amounts of protein were resolved in a 7% Bis–Tris polyacrylamide gel. Proteins were transferred to nitrocellulose membranes (LI‐COR Biosystem). Membranes were blocked using 5% dry nonfat milk in .5% Tween 20 in PBS and then probed using the following antibodies: MLL^N^/HRX (clone N4.4) (Millipore), vinculin (clone EPR8185) (Abcam). Membranes were imaged using Odyssey CLX imaging system (LI‐COR Biosystem).

### 2.9 RNA isolation and qRT‐PCR

Total RNA was isolated using the RNeasy Mini Kit (Qiagen) and RNA concentration was measured using a NanoDrop ND‐1000 (Labtech International). RNA (1 μg) was converted in cDNA using the High Capacity RNA‐to‐cDNA kit (Thermo Fisher) according to manufacturer's instructions. Quantitative RT‐PCR was performed and analysed using the StepOnePlus Real‐Time PCR system (Life Technologies). Relative expression was calculated using the ΔΔCt method, and 18s was used as the reference gene. TaqMan gene expression arrays FAM‐MGB: HOXA10 (Hs00172012_m1), MEIS1 (Hs01017441_m1), c‐MYB (Hs00920556_m1) and 18s (Hs99999901_s1) (all from Thermo Fisher).

### 2.10 Zinc ejection assay

Release of zinc ions from CXXC‐purified protein was monitored by fluorescence emission from the zinc‐specific fluorophore FluoZin‐3 (Invitrogen/Life Technologies). CXXC domain was expressed and purified as described before.^20^ DSF, the zinc ejecting agent, was dissolved in DMSO to a stock solution of 100 mM and then diluted down in PBS pH 7.5. DSF reacted with CXXC protein (5 μM) and FluoZin‐3 (5 μM) in a total volume reaction of 100 μl at room temperature. Fluorescence emission was monitored by FLUOstar OPTIMA (BGM LABTECH) at an excitation wavelength of 494 nm and an emission wavelength of 516 nm.

### 2.11 Library preparation, RNA sequencing and data analysis

Samples were processed using the KAPA mRNA HyperPrep Kit (p/n KK8580) according to manufacturer's instructions. Briefly, mRNA was isolated from total RNA using Oligo dT beads to pull down polyadenylated transcripts. The purified RNA was fragmented using chemical hydrolysis (heat and divalent metal cation) and primed with random hexamers. Strand‐specific first strand cDNA was generated using reverse transcriptase in the presence of Actinomycin D. The second cDNA strand was synthesised using dUTP in place of dTTP to mark the second strand. The resultant cDNA was then ‘A‐tailed’ at the 3′ end. Truncated adaptors, compatible with Illumina sequencing, were ligated to the A‐tailed cDNA. Ligated cDNA molecules were then enriched with limited cycle PCR (10–14 cycles – the actual number was dependent on the amount of input RNA). To make the library strand specific, only the first strand was amplified for sequencing. Libraries were quantified (using Qubit and Bioanalyzer), normalized and pooled before sequencing on a NextSeq 500 (Illumina, San Diego, US) generating 43‐bp read pairs per sample. Run data were demultiplexed and converted to fastq files using Illumina's fastq Conversion Software v2.19. Fastq files were preprocessed to remove adapter contamination and poor‐quality sequences (trimmomatic v0.36) before being mapped to the human genome (GRCh38) using the STAR aligner (v2.5b). Mapped data were deduplicated using Picard Tools (v2.7.1), and the remaining reads per transcript were counted by FeatureCounts (v1.4.6p5). Normalization, modelling and differential expression analysis were then carried out using SARTools (v1.3.2), an integrated QC and DESeq2 BioConductor wrapper. Significant genes were filtered by a desired Benjamini–Hochberg False Discovery Rate and fold change.

### 2.12 Gene set enrichment analysis (GSEA)

Gene expression data were analysed for enrichment using gene set enrichment analysis (GSEA) software (Broad Institute – version 4.1.0). GSEA ranked a list of normalized RNA‐seq data according to the expression difference (signal‐to‐noise ratio) and calculates an enrichment score (ES) by walking down this list and increasing a running sum statistic when it encounters a member within the gene set definition. Conversely, this statistic decreases when encountering a gene not in the gene set. The maximum deviation from zero constitutes the ES and corresponds to a weighted Kolmogorov–Smirnov‐like statistic. Once all gene sets have been evaluated, GSEA adjusts the estimated significance level to account for multiple hypothesis testing and adjusts for the respective sizes of the gene sets, ultimately generating an NES. Use of the NES facilitates comparison across gene sets. The significance (*p*‐value) is also calculated for each NES.

### 2.13 ChIP and ChIP‐seq

For MLL chromatin immunoprecipitation, 25 million cells were crosslinked with disuccinimidyl glutarate 2 mM, incubated for 30 min at room temperature on a rotating wheel and then washed three times with PBS. Subsequently, cell pellets were fixed in formaldehyde 1% for 15 min at room temperature on a rotating wheel and the crosslinking reaction was stopped by adding .125‐M glycine for 5 min at RT. For H3K4me3 and H3K27ac, chromatin immunoprecipitation cells were crosslinked with formaldehyde 1% only. After single or dual crosslink, cells were rinsed twice in cold PBS and cell pellets were suspended in buffer A (Triton X100 .25%, EDTA 10 mM, EGTA .5 mM, HEPES 20 mM pH 7.6). After incubation on rotating wheel at 4°C for 10 min, cells were centrifuged and resuspended in buffer B (NaCl 150 mM, EDTA 10 mM, EGTA .5 mM, HEPES 20 mM pH 7.6). After 10 min on rotating wheel, cells were centrifuged and cell pellet was resuspended in ChIP incubation buffer (SDS .15%, Triton X100 1%, NaCl 150 mM, EDTA 10 mM, EGTA .5 mM, HEPES 20 mM pH 7.6) supplemented with protease inhibitor cocktail (Roche) and incubated for 30 min on ice. Chromatin was sonicated using a Bioruptor Pico sonicator (Diagenode) for 13 cycles, 30‐s ON and 30 s OFF. Sonicated chromatin was centrifuged at 13.200 rpm for 10 min, and supernatant was collected for ChIP. For every ChIP, 900 μl of chromatin (corresponding to 15 × 10^6^) was diluted 1:10 in incubation buffer supplemented with BSA .1%, protease inhibitor cocktail (Roche), 30 μl of protein A beads (Millipore) and 3 μg of antibody (MLL‐N, Diagenode [Catalogue no. C15310265]; H3K4me3 Active Motif [Catalogue no. 39915]; H3K27ac Millipore [Catalogue no. Ab4729]) incubated overnight at 4°C on a rotating wheel. Beads were washed twice with buffer 1 (SDS .1%, sodium deoxycholate .1%, Triton X100 1%, NaCl 150 mM, Tris 10 mM pH 8, EDTA .1 mM, EGTA .5 mM), one time with buffer 2 (SDS .1%, sodium deoxycholate .1%, Triton X100 1%, NaCl 500 mM, Tris 10 mM pH 8.0, EDTA .1 mM, EGTA .5 mM), one time with buffer 3 (LiCl .25 M, sodium deoxycholate .5%, NP40 .5%, Tris 10 mM pH 8.0, EDTA .1 mM, EGTA .5 mM), one time with buffer 4 (Tris 10 mM pH 8.0, EDTA .1 mM, EGTA .5 mM). Chromatin was eluted from the beads using 400 μl of elution buffer (SDS 1%, NaHCO_3_ .1 M) at room temperature for 20 min. Protein–DNA crosslinks were reversed in NaCl 200 mM at 65°C overnight, after which DNA was subjected to RNase and proteinase K digestion for 2 h at 55°C. The DNA was extracted by using the MinElute PCR Purification Kit (Qiagen) and subjected to quantitative PCR (qPCR) using the sensiFAST SYBR Hi‐ROX kit (Bioline) according to manufacturer's instructions. Enrichment in the HOXA10 region was relative to the control gene desert region (gene desert 21, GD21) and was calculated using the per cent input method. Primers sequences were as follows:

*HOXA10* forward: 5′‐ACCGCAGGATGAAACTGAAG‐3′
*HOXA10* reverse: 5′‐TTCCCCCAGAAAACAACAAA‐3′
*GD21* forward: 5′‐GGGGGATCAGATGACAGTAAA‐3′
*GD21* reverse: 5′AATGCCAGCATGGGAAATA‐3′

Samples were processed using the NEB DNA Ultra II Library Kit (p/n E7645) according to manufacturer's instructions. Briefly, 5 ng of ChIP DNA was end‐repaired then ‘A‐tailed’ at the 3′ end to prevent self‐ligation and adapter dimerization. Truncated .6‐μM adaptors, containing a T overhang were ligated to the A‐Tailed cDNA before a ‘double‐SPRI’ bead size selection was used to remove both large (>300 bp) and non‐ligated adaptors (<150 bp). Double ligated DNA molecules were then enriched with limited cycle PCR (12 cycles) utilizing NEB's Q5 high fidelity polymerase. The primers used in this PCR extend the adaptor to full length and contain sequences that allow each library to be uniquely identified by a way of a sample‐specific 6‐bp index sequence. Libraries to be multiplexed in the same run were pooled in equimolar quantities, calculated from the Qubit and Bioanalyzer fragment analysis. Samples were sequenced on the NextSeq 500 instrument (Illumina, San Diego, US) using a 43‐bp paired‐end recipe.

### 2.14 ChIP‐seq analysis

Sequenced reads were aligned against the UCSC human reference genome (GRCh37/hg19) with Burrows–Wheeler Aligner programme with default parameters.^21^ The BAM files were subjected to removal of potential PCR and optical duplicates using Picard MarkDuplicates option. Fragment length and quality measurement for each dataset were determined using PhantomPeakQualTools based on strand cross‐correlation approach. Two metrics named normalized strand cross‐correlation coefficient and relative strand cross‐correlation coefficient were used for data quality assessment. Peak calling was performed using with the estimated fragment size.^22^ Tags within a given region were counted and adjusted to represent the number of tags within a 1‐kb region. Subsequently the percentage of these tags as a measure of the total number of sequenced tags of the sample was calculated and displayed as heat maps using fluff.^23^ To determine genomic locations of binding sites, the peak file was analysed using a script that annotates binding sites according to all RefSeq genes or using GREAT.^24^ MLL dynamic peaks were defined as H3K4me3 peaks (using MACS) with a differential MLL occupancy using a >3‐fold cut‐off.

### 2.15 Animal experiments

All mice were maintained in the animal facilities of the UCL Institute of Child Health and the experiments were performed according to United Kingdom Home Office regulations. Survival analysis was performed using THP‐1‐Luciferase cells treated ex vivo for 6 h with DSF .3 μM in DMSO and CuCl_2_ 1 μM in sterile ddH_2_O or with vehicle only. After treatment, 1 × 10^6^ viable cells were intravenously injected into 5–12‐week‐old NOD.Cg‐Prkdc^scid^Il2rg^tm1Wjl^/SzJ (NSG) mice. To monitor disease progression, mice were imaged every 10 days using the IVIS Lumina Series III (Perkin Elmer) and Kaplan–Meier survival curves were plotted.

DSF in vivo efficacy was studied in NSG mice engrafted each with 1 × 10^6^ viable THP‐1‐Luciferase cells.^25^ Mice were imaged once a week to detect leukaemia engraftment. After engraftment was confirmed, mice were randomly divided in three groups: one control group and two treated groups (group receiving oral treatment and group receiving intraperitoneal [i.p.] injection). The group of mice treated orally was feed with powdered chow mixed with .05% DSF. Each day the mix diet/DSF was prepared fresh. A second group of mice received daily a solution of DSF 5 mg/kg in corn oil via i.p. injection. Copper was resuspended in saline at .5 mg/kg and injected i.p. daily in both treated groups. All mice were treated for 5 days/week for 2 weeks of total treatment. During treatment, mice were imaged once a week in order to assess disease progression.

MLL–ENL immortalized murine leukaemic cells (1 × 10^5^ cells/mouse) were transplanted in sublethally Y‐irradiated C57BL/6J‐CD45.1. Peripheral blood was analysed weekly for the presence of leukaemic cells. As soon as engraftment was confirmed, mice were randomly divided in control and treated groups. The group of treated mice received daily copper and DSF via i.p. injection. DSF was prepared in corn oil at 5 mg/kg and copper in saline at .5 mg/kg. During treatment, peripheral blood was still analysed weekly to monitor disease progression and the expression of the myeloid differentiation marker CD11b. In all in vivo experiments, the animals were sacrificed as soon as they started to show the first signs of leukaemia.

### 2.16 Liquid chromatography–mass spectrometry

Blood samples were collected from NSG mice following 4 weeks of DSF administration (0, 150 or 200 mg/kg) 1 h after the final dose. For each dose, blood samples from three mice were pooled, centrifuged at 2000 rpm for 10 min and plasma collected. Methanol was added to plasma samples in a 1:5 ratio, centrifuged at 2000 rpm for 10 min and supernatant collected. Supernatants were analysed by mass spectrometry at the University of New South Wales (Randwick, NSW, Australia). Samples were injected onto a BEH C18 2.1 × 50‐mm^2^ column (Waters) using a CTC PAL autosampler, and chromatography was performed on an Accela system (Thermo Fisher Scientific) at 400 μl/min. Solvent A was .1% formic acid in water and Solvent B was .1% formic acid in acetonitrile. Gradient separation was as follows: time (min), %B: 0, 0; .5, 0; 3.5, 100; 4.5, 100; 5, 0; 7, 0. Column eluates were directed into the heated electrospray source of a quantum access mass spectrometer (Thermo Fisher Scientific). SRM transitions selected were 297 > 116 @11V (for quantification), 297 > 148 @16V and 297 to 88 @25V (for confirmation). Data were processed and chromatograms integrated automatically using Xcalibur software.

## 3 RESULTS

### 3.1 A screening platform for MLL‐fusion protein depletion

**FIGURE 1:**
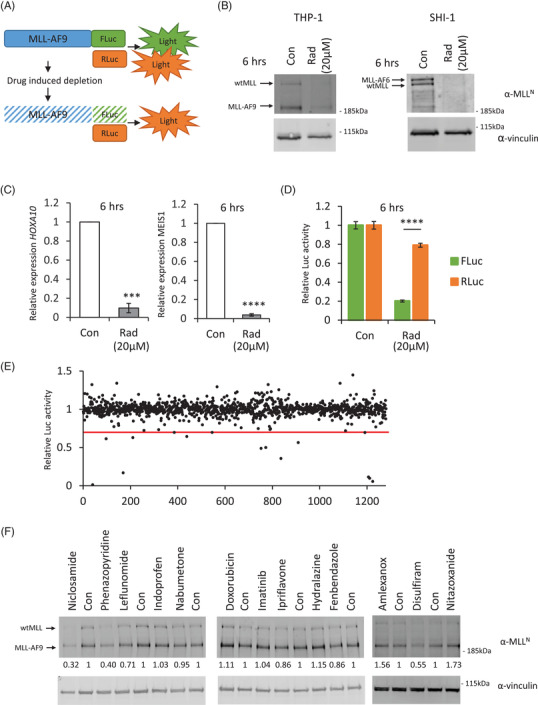
MLL‐fusion screening platform: (A) schematic overview of the cellular MLL‐fusion depletion assay. MLL‐AF9 was fused to the firefly luciferase gene (FLuc) and cloned into the pMSCV retroviral backbone, whereas renilla luciferase was cloned without any modifications into the pMSCV backbone. The principle of the assay involves measuring FLuc/RLuc activity correlating to normalized MLL‐AF9 protein levels. (B) Radicicol depletes both wild‐type MLL and MLL‐fusion proteins. Human leukaemic cells THP‐1 and SHI‐1 expressing either MLL‐AF9 or MLL‐AF6, respectively, were treated for 6 h with 20 μM of radicicol. Total cellular lysates were analysed by western blotting. Wild‐type MLL and MLL‐fusion proteins were specifically depleted. No effect on vinculin was observed. (C) Effects of MLL‐fusion depletion by radicicol on target gene expression in *MLL*‐rearranged SHI‐1 cells. The expression of *HOXA10* and *MEIS1* were examined through RT‐quantitative PCR (qPCR)  6 h after 20‐μM radicicol treatment. The relative expression was normalized to 18 s. Bar charts are mean ± SD (one sample *t*‐test, *p < .05, **p < .01, ***p < .001) of three independent experiments. (D) Quantification of FLuc and RLuc activity upon 6‐h treatment with 20‐μM radicicol in cells co‐transduced with the MLL‐AF9‐FLuc and RLuc. The FLuc activity is specifically reduced showing that FLuc activity is dependent on the MLL‐fusion protein level. (E) The MLL‐fusion depletion screen was performed using the Prestwick clinical compound library. Cells were treated with 10 μM of each compound for 6 h, and MLL‐fusion levels were measured using a dual‐luciferase assay. Normalized FLuc/RLuc ratios are plotted. Thirteen compounds showed a reduction larger than 33% in normalized FLuc activity. (F) Hit verification by western blotting. MLL‐AF9 expressing cells (THP‐1) were treated with the indicated compounds at 10 μM for 6 h. Total cellular lysates were analysed by western blotting. Three compounds showed a reduction larger than 33% in normalized MLL‐fusion levels

To address the urgent need for new therapeutics for *MLL*‐rearranged leukaemias, we generated a bioluminescence‐engineered MLL‐fusion reporter cell line, called THP1‐MA‐Luc, in which the ratio of FLuc/RLuc is used as a measure of the amount of MLL‐AF9 in these cells (Figure 1A). Radicicol, a natural product that is too unstable for in vivo use, has previously been described to deplete the endogenous non‐rearranged MLL.^26^, ^27^ We showed that radicicol depletes both the endogenous non‐rearranged MLL and MLL‐fusion proteins (Figure 1B). This decrease in MLL‐fusion protein resulted in silencing of its target genes *HOXA10* and *MEIS1* (Figure 1C).^28^, ^29^ We confirmed that the decrease in FLuc activity correlated with reduced protein level after radicicol treatment (Figure 1D). The quality control parameter Z′ factor calculated for this reporter system was .72, indicating an excellent assay.^30^ Thus, this experimental setting was suitable for the screening and identification of drugs that deplete the MLL‐fusion proteins.

The THP1‐MA‐Luc cell line was used for a primary screening of the Prestwick Chemical library, a collection of 1280 off‐patent small molecules most of which FDA approved, at a similar concentration and duration of treatment as the Connectivity Map (10 μM, 6 h).^31^, ^32^ Compounds that were clearly toxic in our screen, defined as >50% in RLuc activity, were excluded (56 compounds). Thirteen compounds met the criterion for an active compound, defined as a ≥33% reduction in the normalized FLuc/RLuc ratio (Figure 1E, Table S1). These compounds were then retested by western blotting for endogenous MLL‐AF9 (Figure 1F). Three of the compounds showed a decrease >33% in the secondary screen.

### 3.2 Disulfiram treatment reduces MLL‐fusion proteins in leukaemic cells

**FIGURE 2:**
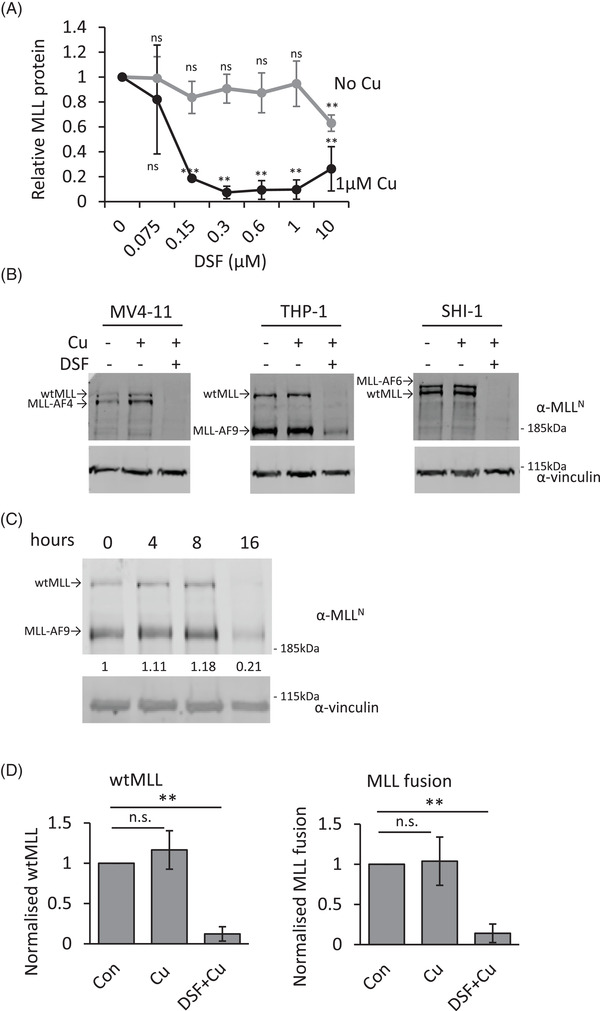
Effect of disulfiram (DSF) treatment on MLL wild‐type and MLL‐fusion proteins levels. (A) Western blot analysis of MLL‐fusion proteins. Protein samples from *MLL*‐rearranged cells treated with the indicated concentrations with or without the supplementation of 1‐μM Cu for 16 h. The proteins were detected using antibodies against N‐terminal MLL and vinculin. The quantification of the blots, normalized to vinculin levels, is plotted. Error bars represent SD from three cell lines expressing either MLL‐AF4, MLL‐AF9 or MLL‐AF9. (B) Representative western blot of MLL‐depletion by DSF and Cu. (C) Time course of MLL‐AF9 expressing THP‐1 cells incubated with .3‐μM DSF and 1‐μM Cu. The proteins were detected using antibodies against N‐terminal MLL and vinculin. (D) Quantification of MLL‐fusions and wild‐type MLL normalized to vinculin. The error bars represent SD of three independent experiments. Bar charts are mean ± SD (*t*‐test, *p < .05, **p < .01, ***p < .001)

We focused our attention on DSF, used in the management of chronic alcoholism, as it is well tolerated and has a clear efficacy in cancer patients.^33^, ^34^, ^35^ Furthermore, it was shown that DSF is potentiated upon copper chelation (forming the active diethyldithiocarbamate [DDC]–copper complex).^33^ Unlike human serum, fetal bovine serum is almost completely devoid of Cu.^36^ We therefore supplemented our media with 1 μM of Cu, equating to ∼50% of the free Cu in human serum.^37^ A six‐point dose–response curve was generated for endogenous MLL‐AF9, MLL‐AF4 and MLL‐AF6 with or without Cu, in different AML cell lines (Figures 2A and S1A–C). A volume of 300‐nM DSF supplemented with Cu almost completely depleted the fusion protein (Figure 2B). We confirmed that the metabolite DDC could also ablate MLL‐fusion protein (Figure S1D). Time course experiments showed that, upon drug treatment, the MLL protein had declined at 16 h (Figure 2C). It is interesting to note that DSF ablates both the MLL‐fusion protein as well as the wild‐type protein to a similar degree (Figure 2D).

### 3.3 Disulfiram blocks the oncogenic program in *MLL*‐rearranged leukaemias

**FIGURE 3:**
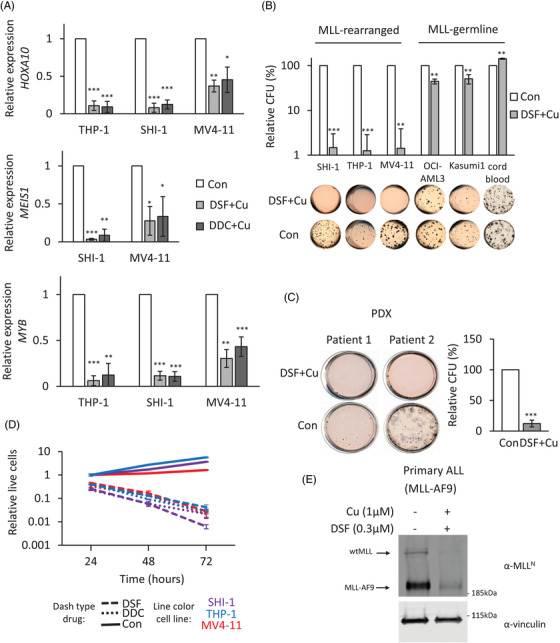
Effects of MLL‐fusion depletion by disulfiram (DSF) treatment. (A) Effects of DSF–Cu on target gene expression in *MLL*‐rearranged cells. The expressions of *HOXA10*, *MEIS1* and *MYB* were examined after 16 h with .3‐μM DSF or .6‐μM diethyldithiocarbamate (DDC) in the presence of 1 μM of Cu treatment through RT‐quantitative PCR (qPCR). The relative expression was normalized to 18 s. Bar charts are mean ± SD (*t*‐test, *p < .05, **p < .01, ***p < .001) from three independent experiments. (B) CFU assay of acute myeloid leukaemia (AML) or CD34^+^ cord blood cells treated with DMSO or .3‐μM DSF in the presence of 1 μM of Cu. Data represent the mean of three independent experiments. Bar charts are mean ± SD (C) CFU assay of two *MLL*‐rearranged patient–derived xenograft samples treated with DMSO or .3‐μM DSF in the presence of 1 μM of Cu. Data represent the mean of two independent patient samples repeated twice. Bar charts are mean ± SD and *t*‐test performed. (D) Anti‐proliferative activity of DSF and DDC against *MLL*‐rearranged AML. Cells were exposed to DMSO, DSF (.3 μM) or DDC (.6 μM) supplemented with 1 μM of Cu for periods varying from 0 to 72 h. Data illustrate a representative experiment of three independent replicates. Error bars are ±SD (E) western blot analysis of DSF effect on patient derived ALL MLL‐AF9 cells. MLL‐AF9 ALL cells were treated with .3‐μM DSF and 1‐μM Cu for 16 h. The proteins were detected using antibodies against N‐terminal MLL and vinculin

Inactivation of the MLL‐fusion protein would be expected to shut down the cellular machinery driving these leukaemias. It is well established that MLL‐fusions induce aberrant upregulation of their target genes, *HOXA10*, *MEIS1* and *c‐MYB*.^28^, ^29^ DSF treatment resulted in the downregulation of these MLL‐fusion target genes (Figure 3A). (MEIS1 is not expressed nor bound by MLL‐AF9 in THP‐1 cells.^29^) Inactivation of MLL‐fusion protein should result in a block in colony formation.^16^, ^38^ Although DSF did not reduce the colony formation of CD34^+^ cord blood cells, DSF could block the colony formation ability of *MLL‐*rearranged cell lines. In non‐*MLL*‐rearranged cell lines, DSF showed a smaller but still significant reduction in colony formation which is mostly likely due to the inhibition of other DSF targets, for example, aldehyde dehydrogenase ALDH^39^ (Figure 3B). The block in colony formation by DSF was confirmed in two *MLL*‐rearranged patient‐derived xenograft samples (Figure 3C). In liquid cultures, DSF and DDC treatment on *MLL*‐rearranged cell lines resulted in a block in the growth and induction of cell death (Figures 3D and S2A–C). DSF was also able to ablate MLL‐fusion protein in a primary *MLL*‐rearranged ALL sample (Figures 3E and S2D). Furthermore, it was able to block the growth of primary *MLL*‐rearranged ALL cells in a human mesenchymal stem cell coculture^40^ (Figure S2E,F). Taken together, these data indicate that DSF can inactivate MLL‐fusion proteins, resulting in down‐regulation of their target genes, leading to a block in colony formation and block in cell growth, resulting in cell death.

### 3.4 Disulfiram abolishes DNA binding activity of MLL‐fusion proteins

**FIGURE 4:**
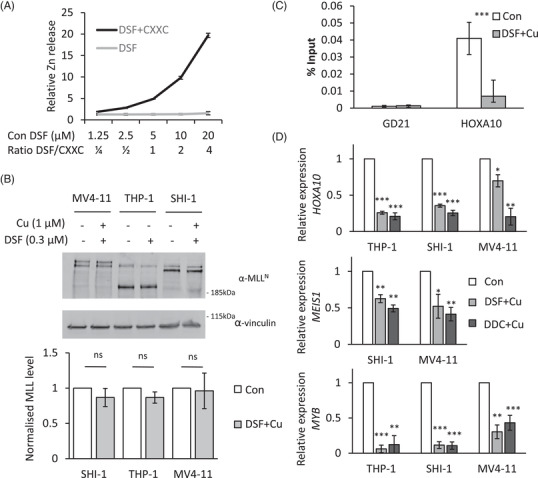
Disulfiram (DSF) targets the CXXC DNA binding domain of MLL fusion proteins. (A) Recombinant MLL CXXC domain protein was incubated with DSF, and the release of zinc ions from MLL CXXC domain was monitored by the fluorescence signal of the Zn‐specific fluorophore, FluoZin‐3. (B) Western blot analysis of MLL‐fusion proteins. Protein samples from *MLL*‐rearranged cells treated with .3‐μM DSF supplemented with 1‐μM Cu for 4 h. The proteins were detected using antibodies against N‐terminal MLL and vinculin. The quantification of the blots, normalized to vinculin levels, is plotted. Error bars represent SD from three independent experiments on cells expressing MLL‐AF4, MLL‐AF9 and MLL‐AF9. Bar charts are mean ± SD (C) chromatin immunoprecipitation (ChIP)‐quantitative PCR (qPCR) analysis of MLL at the HOXA10 locus. A gene desert on chromosome 21 was used as a negative control. Data are normalized to per cent input. Significant differences were observed among the 4‐h DSF treated cells compared to control. Data are presented as mean ± SD of four independent experiments. ***p < (D) The expressions of *HOXA10*, *MEIS1* and *MYB* were examined through RT‐qPCR  4 h after DSF‐Cu or diethyldithiocarbamate (DDC)‐Cu treatment. The relative expression was normalized to 18 s. Bar charts are mean ± SD (*t*‐test, *p < .05, **p < .01, ***p < .001) of three independent experiments.

DSF has previously been identified as a specific inhibitor of the CXXC domain (C is cysteine; X is any other amino acid), through the release of zinc from this domain.^41^, ^42^ The MLL CXXC DNA‐binding domain contains a specific clustering of eight cysteines which coordinate two zinc ions.^20^, ^43^, ^44^ This would suggest that DSF could inactivate the DNA binding activity of MLL‐fusion proteins by disrupting the structural CXXC domain. This would prevent binding to its target sites and lead, at a later timepoint, to the destabilization of the MLL‐fusion protein that we have detected. To test this hypothesis, we used the purified recombinant MLL CXXC domain (residues T1136–K1208) to establish if DSF was able to eject the zinc from this domain.^20^ As shown in Figure 4A, an addition of DSF to the recombinant MLL CXXC domain resulted in the release of Zn^2+^ ions, as detected by changes in fluorescence of the zinc‐specific fluorophore FluoZin‐3. The CXXC domain is essential for target gene recognition, transactivation and myeloid transformation by MLL‐fusion proteins.^44^ As we hypothesized, the ejection of zinc should therefore prevent the binding of MLL‐fusions to their target genes before the protein gets ablated. We treated *MLL*‐rearranged cells for 4 h and could not detect a significant change in the protein levels and assessed, using an N‐terminal MLL antibody, the binding of MLL to the *HOXA10* promoter (Figure 4B,C). This chromatin immunoprecipitation (ChIP)‐qPCR experiment showed a strong reduction in the binding of the MLL protein to the *HOXA10* promoter. It indicates that the fusion protein had lost its target gene recognition ability. Finally, an active CXXC domain is essential for the expression of the MLL‐fusion targets. Inactivation of the MLL‐fusion proteins by DSF reduced the expression of the MLL target genes within 4 h of treatment (4D). It confirms that the fusion protein had lost its ability to transactivate these genes. In conclusion, these findings suggest that DSF inactivates the CXXC domain and blocks MLL binding to its loci. This leads to structural changes of these loci resulting in silencing of key pathways that drive transformation by MLL‐fusion proteins.

### 3.5 Disulfiram blocks leukaemic stem cell activity and reduces leukaemic burden

**FIGURE 5:**
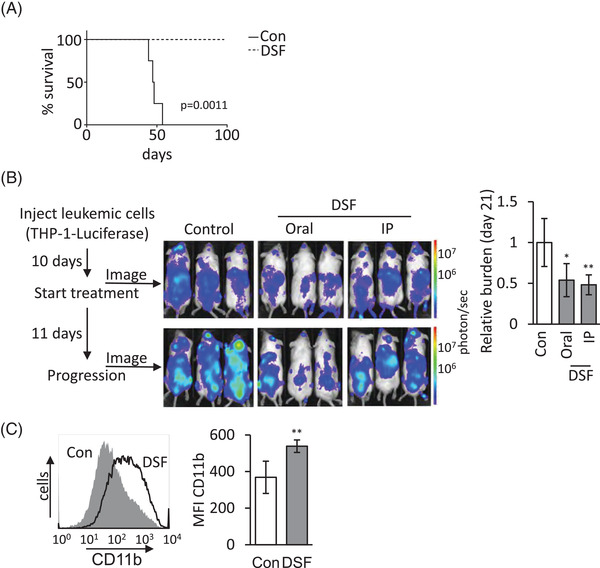
Disulfiram (DSF) blocks leukaemic stem cell activity and reduces leukaemic burden in vivo. (A) DSF treatment blocks the engraftment of *MLL*‐rearranged leukaemia. A million MLL‐AF9 rearranged THP‐1‐luciferase cells either treated with DSF/Cu or vehicle control for 16 h were injected intravenous (i.v.) into NSG mice. Kaplan–Meier survival curves in each group as indicated (*n*  =  6 for Con and *n* = 4 for DSF). (B) Outline of treatment of *MLL*‐rearranged acute myeloid leukaemia (AML) with DSF. NSG mice (*n* = 6 mice/group) were either orally or intraperitoneal (i.p.) treated with DSF or vehicle 10 days after i.v. injection of THP‐1‐luciferase cells (5 × 10^5^ cells/mouse). DSF was subsequently administered 5 days/week. Leukaemia engraftment was tracked by serial luciferase measurements. Examples and quantification of leukaemic burden shows a statistically significant difference for both oral and i.p. administration of DSF as indicated (*p < .05; **p < .001). (C) C57BL/6J mice (*n* = 5 mice/group) were either i.p. treated with DSF or vehicle 2 days after i.v. injection of murine MLL–ENL overexpressing leukaemia (1 × 10^5^ cells/mouse). DSF was subsequently administered for 5 days and CD11b expression was assessed by flow cytometry. Example and quantification show a statistically significant up‐regulation of CD11b expression (*p* < .001).

Leukaemic stem cell activity is defined by the capacity to engraft and reproduce disease in transplants.^45^ To test whether MLL‐fusion ablation could affect leukaemia engraftment and leukaemia‐initiating capacity, we used a luciferase‐labelled THP‐1 cells for our AML xenograft model.^25^ We examined the effect of DSF treatment on leukaemic cell engraftment capacity after 16 h DSF ex vivo exposure. Leukaemic engraftment and/or homing was clearly inhibited by DSF treatment, as evidenced by an absence of luciferase signal (Figure S3A). All the vehicle‐treated recipients succumbed to lethal leukaemia (maximum survival 55 days), whereas no disease occurrence was observed in DSF‐treated group monitored up to 100 days (Figure 5A). These results indicate that DSF treatment diminished leukaemic engraftment and initiating capacity. Next, we evaluated the efficacy of in vivo administration of DSF. Previous studies have noted that the concentrations of DSF in the serum of mice of DSF are an order of magnitude lower compared to the levels in humans undergoing DSF treatment for alcoholism.^33^ Population pharmacokinetics and pharmacodynamics studies of DSF have shown that a dose of ∼33 mg/kg for three consecutive days resulted in a DSF plasma concentration of .3–1.7 μM between 0 and 2 h after the last dose.^46^ This was in stark contrast to mice that, after 4 weeks (5×/week) of up to 200‐mg/kg DSF treatment, showed a plasma concentration of DSF in the picomole ranges, ∼1000‐fold below the 10‐nM spike in, 1 h after the last dose (Figure S3B). Despite this, we generated cohorts of leukaemic‐bearing mice, by transplanting luciferase‐labelled THP‐1 cells. After 10 days, leukaemia‐bearing mice were treated with only Cu (via i.p. injection) or Cu (i.p.) and DSF mixed in the chow or Cu and DSF via i.p. injections. We found that the leukaemic burden was effectively reduced, compared to control, 11 days after initiating DSF treatment (Figure 5B). We noted no difference for different routes of administration. To further evaluate the efficacy of DSF treatment, we engrafted mice with a mouse leukaemic MLL‐fusion previously shown to differentiate upon loss of MLL‐fusion protein as measured by CD11b expression.^38^ Mice treated with DSF showed a significant up‐regulation of CD11b expression on the leukaemic blasts compared to control‐treated mice (Figure 5C).

### 3.6 DSF induces MLL‐fusion‐dependent dynamic epigenetic changes

**FIGURE 6:**
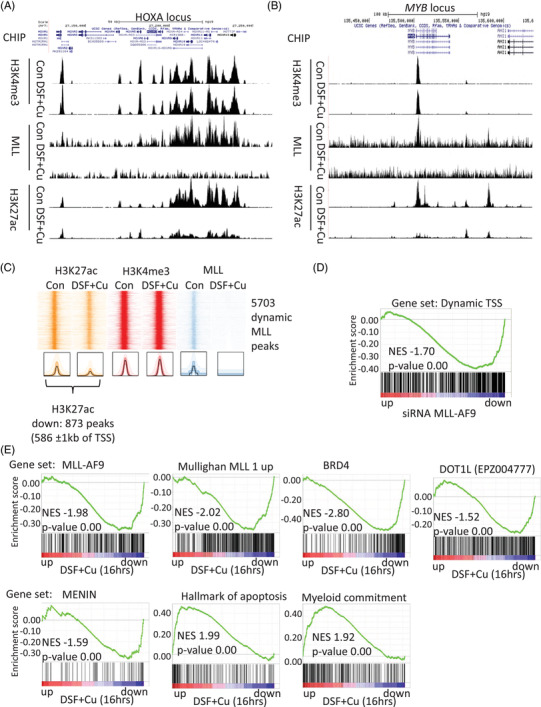
Global chromatin immunoprecipitation (ChIP)‐seq changes and functional consequences of disulfiram (DSF) treatment. (A and B) UCSC genomics viewer snapshots of ChIP‐seq tracks for H3K4me3, MLL and H3K27ac in 4‐h DSF or control‐treated THP‐1 cells aligned over the HOXA cluster and MYB gene. (C) Heat map with ChIP‐seq profile (top) of H3K27ac, H3K4me3 and MLL signal around peak centre at MLL peaks (5 kb on both sides of the peak) within control sample with 4 h of DSF or control treatment in THP‐1 cells. ChIP‐seq analysis identifies 873 dynamic MLL/H3K27ac peaks of which 586 located within 1 kb of transcription start site (TSS). (D) Gene set enrichment analysis (GSEA) analysis of genes with dynamic MLL binding at TSS among genes ranked according to fold change in expression following knockdown of MLL‐AF9 in THP‐1 acute myeloid leukaemia (AML) cells. (E) GSEA plots show negative enrichment of gene sets regulated by MLL‐fusion and enrichment for myeloid differentiation and early apoptosis among genes ranked according to fold change in expression following treatment of THP‐1 AML cells with 300‐nm DSF and 1‐μM Cu for 16 h

Epigenetic reprogramming plays an instrumental role in MLL‐fusion transformation. ChIP‐seq analysis was utilized to further investigate the binding profile of MLL and the related epigenetic changes 4‐h post‐DSF treatment. ChIP‐seq analysis showed no binding of MLL on HOXA and MYB locus after 4 h of DSF treatment (Figure 6A,B). We observed that 4‐h DSF treatment resulted in no significant changes in H3K4me3 levels at the *HOXA* and *MYB* locus (Figure 6A,B). This contrasted with the MLL and H3K27ac regions which both showed a strong reduction upon DSF treatment. These data match the much slower dynamics of H3K4me3 (*t*1/2 = 6.8 h) compared to H3K27ac (*t*1/2 = 1.1 h) turnover.^47^, ^48^ The global ChIP‐seq analyses showed 5703 dynamic MLL regions (with greater than threefold change in signal) marked by no changes in H3K4me3 (Figure 6C). These regions correlated with previously identified MLL‐fusion binding sites (Figure S4A).^29^, ^49^ All 5703 regions showed co‐localization with H3K27ac, which were threefold reduced at 873 and increased at 18 regions. GREAT analysis of the reduced MLL/H3K27ac peaks associated with these regions with 586 peaks at promoter regions (defined as ±1 kb of a transcription start site [TSS]).^24^ These promoter regions (named Dynamic TSS) were enriched for genes that are down‐regulated upon MLL‐AF9 knockdown, in other words, the Dynamic TSS colocalize with genes transactivated by MLL‐fusion proteins (Figure 6D).^50^ Furthermore, reduced MLL/H3K27ac associated genes were strongly enriched in the MLL signatures identified by Mullighan et al. (Figure S4B).^51^ To confirm the functional consequences of changes seen in the ChIP‐seq data, we generated expression profiles using genome‐wide transcriptomic (RNA‐seq) analysis of DSF‐treated THP‐1 cells. The DSF‐down‐regulated genes were negatively enriched for published MLL‐fusion signatures, including those generated by MLL‐AF9 siRNA‐mediated silencing (*p* ≤ .01, unpaired *t*‐test and at least mean twofold decrease in expression) and the Mullighan et al. signatures (Figure 6E).^50^, ^51^ BRD4, MENIN and DOT1L regulate MLL‐fusion‐dependent gene pathways. GSEA analysis of the genesets of signatures of BRD4 (human homologues of Brd4 knockdown with *p* ≤ .01, unpaired *t*‐test and at least mean twofold decrease in expression),^52^ MENIN (CRISPR knockout with *p* ≤ .01, unpaired *t*‐test and at least mean twofold decrease in expression)^53^ and DOT1L inhibitor (EPZ004777 treatment of MOLM‐13 with *p* ≤ .01, unpaired *t*‐test and at least mean log twofold decrease in expression)^17^ all showed negative enrichment indicating that DSF interferes with these important signalling pathways in *MLL*‐rearranged leukaemias. Furthermore, the DSF‐induced gene expression changes showed positive enrichment for myeloid commitment, indicating up‐regulation of myeloid differentiation programme, and hallmarks of apoptosis^54^ (Figure 6E). This reinforced the data from our functional experiments.

## 4 DISCUSSION

Fusion proteins have long been recognized as driver events in leukaemia. They are specific to the leukaemic cells, and, therefore, constitute an exceptional target. However, a traditional chemical design of fusion protein inhibitors has proven challenging as many of these molecules do not contain traditional ‘druggable’ pockets. Here, we present a protein depletion screen coupled with drug repurposing as a novel avenue in anti‐cancer drug development to overcome the traditional limitations to targeting transcription factors. We demonstrate the value of this approach through the identification of a novel therapy for *MLL*‐rearranged leukaemias.

The activity of MLL1 is, at least in part, regulated through its proteolytic turnover.^27^, ^55^, ^56^ Although the regulatory proteolysis of MLL1‐fusion proteins is disrupted, we show here that the turnover regulated by HSP90 is maintained for both the wild‐type and fusion proteins. Inhibition with HSP90 inhibitor radicicol resulted in a depletion of MLL fusion and a down‐regulation of MLL‐fusion target genes. Radicicol is too unstable for clinical use.^26^ Therefore, we designed a mechanistic screening approach that would identify small molecules which decrease MLL‐fusion protein levels. This screening approach allowed us to validate three bioactive compounds which could down‐regulate MLL‐fusion protein by more than 33%. We focused our attention to DSF as previous studies have shown that DSF is a potent anti‐cancer agent that can affect protein degradation/turnover when combined with Cu(II) ions.^33^, ^57^, ^58^, ^59^ Adding free Cu, equating about 50% of free Cu in human serum, greatly enhanced the activity of DSF and almost completely depleted MLL wild‐type and fusion proteins. This led to a block in colony formation exclusively to the *MLL*‐rearranged models. Previous studies have shown that wild‐type MLL, while being essential during embryogenesis, is dispensable for the production of haematopoietic lineages.^60^ In concordance, the DSF treatment of cord blood did not block colony formation. Wild‐type MLL has been shown to play an important role in some solid cancers, and we have started to explore the possibility of utilizing DSF in solid cancers.^61^

MLL‐fusion proteins support self‐renewal activity via aberrant transcriptional activation of master regulators (e.g. *HOXA10*, *MEIS1* and *MYB*), confer resistance to apoptosis and repress differentiation programmes. DSF inhibited the expression of those stemness‐associated master regulators, and stem cell activity was abolished as treated cells had lost the ability to engraft in recipient mice. Our data, together with previously published results, have indicated significant lower plasma levels of DSF in mouse models compared to the levels achieved in humans despite giving higher doses of the drug. Despite achieving very low levels of DSF in our model systems, we could show not only a reduction in the leukaemic burden but also a triggered expression of a differentiation‐associated programme.

The oncogenic activity of the MLL‐fusion protein is dependent on the DNA binding activity of the CXXC domain to the promoters and/or enhancers of its target genes.^20^, ^44^, ^62^, ^63^ DSF contains strong thiol‐reactive functional groups that have been shown to disrupt CXXC domains.^42^, ^59^ Disruption of these structural zinc containing domains can lead to a loss in protein activity and protein unfolding.^64^ Here we have shown that DSF was able to ablate the activity of the MLL‐fusion protein through blocking the DNA binding of MLL‐fusion proteins. This led to the down‐regulation of its target genes, including the stemness‐associated master regulators *HOXA10*, *MEIS1* and *MYB*. These rapid initial changes in DNA binding activity were followed by the depletion of the fusion protein from the leukaemic cells.

MLL‐fusion proteins bind to their target loci recruiting various histone‐modifying complexes leading to an increase in H3K4me3 and H3K27Ac marks and augmented expression.^29^, ^65^

To uncover the epigenetic mechanisms underlying rapid transcriptional changes occurring upon MLL fusion inactivation, we analysed genome‐wide dynamics of the chromatin landscape associated with MLL fusion associate histone marks H3K4me3 and H3K27ac. Particularly, our analysis highlights a prominent role for histone acetylation in facilitating transcriptional response upon MLL inactivation by DSF, as these correlated with genes that are silenced upon MLL‐AF9 knockdown. In contrast to histone acetylation, H3K4me3 did not change at this early time point which can be explained by the slow turnover rate of pre‐existing H3K4me3 methylation. DSF treatment of MLL‐rearranged leukaemia cells induces overall very similar transcriptional changes as a knockdown of the fusion protein and negatively correlated with the genes up‐regulated in pediatric *MLL*‐rearranged AML, as described by Mullighan et al.^51^

We have validated a novel and robust screening approach to uncover drugs that can deplete MLL‐fusion proteins and characterized DSF as a novel therapy for *MLL*‐rearranged leukaemias. DSF has great potential as a treatment for MLL‐fusion‐driven leukaemias due to its known drug profile in humans, low‐cost, little side effects and selectivity against *MLL*‐rearranged leukaemic cells.^46^, ^66^ Besides inhibiting the H3K4me3 writer protein MLL DSF has also been shown to inhibit H3K4me3 reader proteins, including the oncogenic fusion protein NUP98‐PHF23 (NP23).^67^ NP23, like MLL‐fusions, activates the expression of *HoxA* cluster and *MEIS1*. Inv(11)(p15q23), found in myelodysplastic syndromes and AML, leads to the expression of a fusion protein consisting of the N‐terminal of nucleoporin 98 (NUP98) and the majority of MLL1. DSF has therefore the potential to be in interesting candidate to study in this and other leukaemias characterized by high *HoxA* cluster and *MEIS1* expression. Our data support the proposed essential role of MLL fusions in leukaemogenesis. DSF and small molecular inhibitors for DOT1L,^17^ BRD4^18^ and MENIN^19^ that target MLL fusion–dependent gene pathways have the potential to radically change the outcome of patients with *MLL* rearrangements.

## 5 CONCLUSIONS

The MLL family of oncogenic fusion proteins are aberrant transcriptional regulators that play a prominent oncogenic role in a variety of leukaemias. Although downstream pathways of this oncoprotein have already been targeted through several therapeutic strategies, this study is the first to present data on the direct inhibition and degradation of these oncogenic drivers. This reveals previously unrecognized therapeutic opportunities that should be vigorously pursued. As DSF has widely been used in the treatment of alcohol dependency, the pharmacological characteristics are well studied. This places DSF in an optimal position for inclusion in clinical trials in the near future to assess its therapeutic potential.

## CONFLICT OF INTEREST

The authors declare that there is no conflict of interest that could be perceived as prejudicing the impartiality of the research reported.

## Supporting information

Supporting Information
