# Supplementary material for: Direct targeted therapy for MLL‐fusion‐driven high‐risk acute leukaemias
Source: Clin Transl Med. 2022 Jun 22;12(6):e933. doi: 10.1002/ctm2.933 (PMC9214753; doi:10.1002/ctm2.933)
Supplement: Supplementary file 1 — Supporting Information [file CTM2-12-e933-s001.pdf]

Supplementary table 1

Chemical name	Fluc/Rluc ratio
Control	1.00
Niclosamide	0.01
Disulfiram	0.61
Hydralazine hydrochloride	0.17
Fenbendazole	0.63
Doxorubicin hydrochloride	0.64
Phenazopyridine hydrochloride	0.49
Leflunomide	0.50
Indoprofen	0.36
Nabumetone	0.56
Amlexanox	0.11
Ipriflavone	0.10
Nitazoxanide	0.06

Lucefirase/Renilla ratio from compounds of positive hits from figure 1E.

Supplementary Figures:

Figure S1

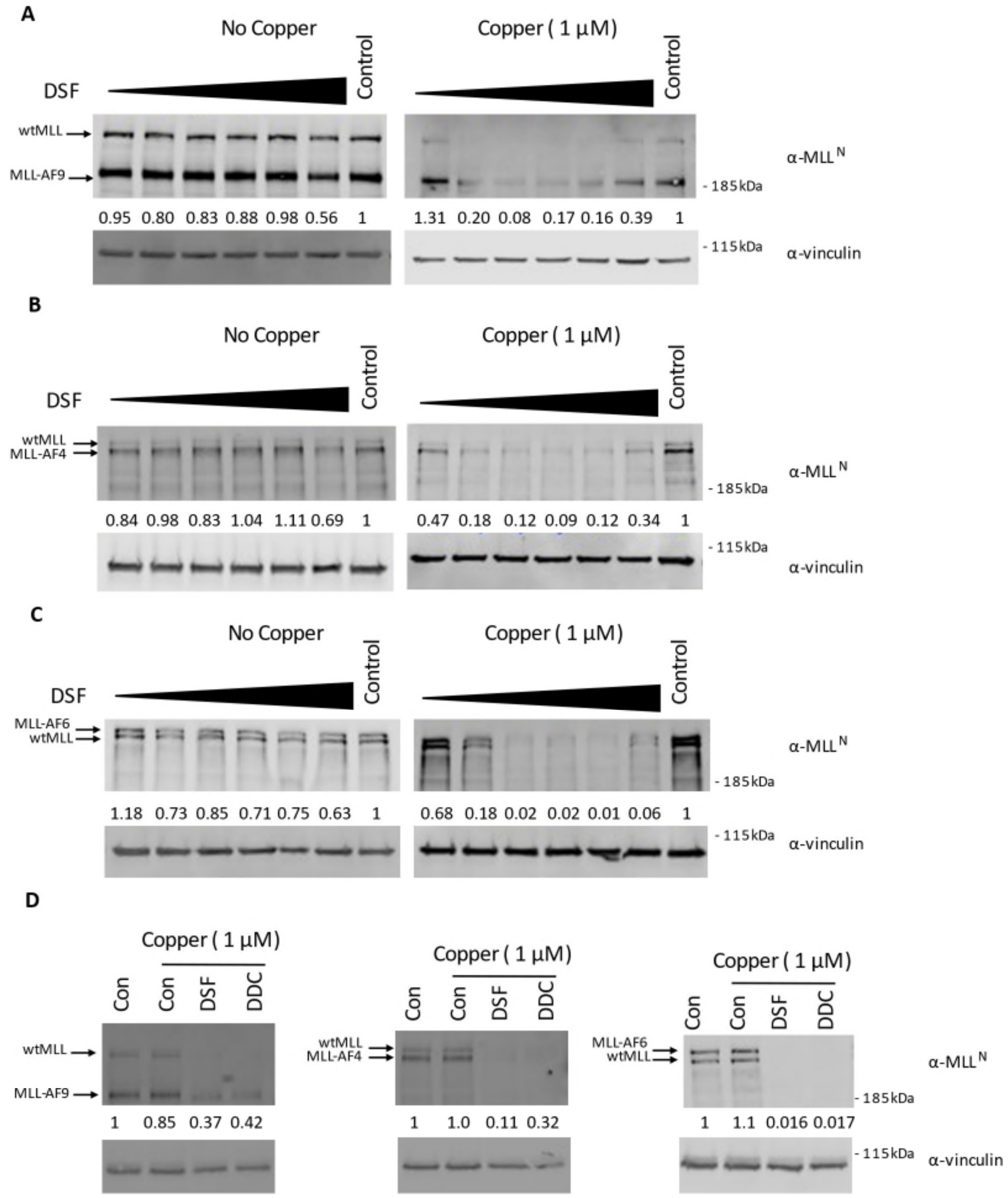

	Raw MLL Signal		
	MV4-11	THP-1	SHI-1
Con	166	895	584
Cu	261	988	663
DSF +Cu	6.85	194	2.2

	Raw Vinculin Signal		
	MV4-11	THP-1	SHI-1
Con	9800	10800	11000
Cu	11800	14600	10500
DSF +Cu	11600	9490	11400

Fig. S1. DSF-copper combination inactivates MLL-fusions and wild-type MLL

Western blot analysis of MLL-fusion proteins. (**A-B-C**) Protein samples from *MLL* rearranged cells treated with the increasing concentrations of DSF with or without the supplementation of 1µM Cu for 16h. The proteins were detected using antibodies against the N-terminal MLL and vinculin. Numbers underneath each lane represent normalized densitometric quantification. (**D**) Protein samples from *MLL* rearranged cells treated with 0.3µM DSF or 0.6µM DDC and supplemented with 1µM Cu treated for 16h. Tables show raw densitometer data from the above western blots.

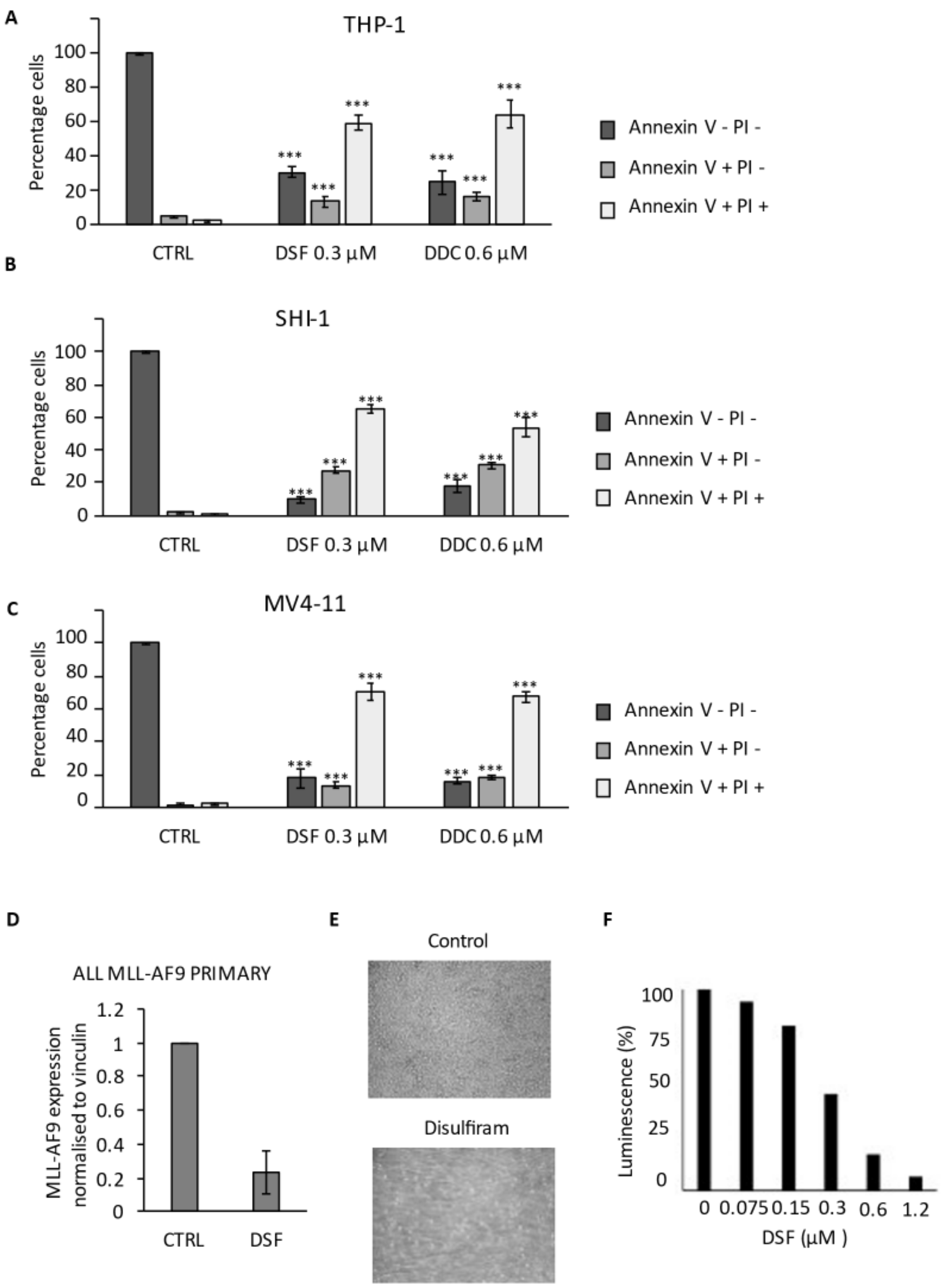

Fig. S2. DSF-copper effect on cell death and primary samples.

(**A-B-C**) DSF and DDC induce cell death after 48h of treatment. MLL-AF9, MLL-AF6, MLL-AF4-expressing cells were treated with 0.3µM DSF or with 0.6µM DDC and both

supplemented with 1µM Cu for 48h. Annexin-PI staining was performed and flow cytometry analysis resulted were plotted. The error bars represent SEM of three independent experiments. Bar charts are mean±s.d. Significant changes versus equivalent control population is indicated (t-test, *: P<0.05, **: P<0.01, ***: P<0.001). (**D**) Quantification of Western blot analysis of DSF effect on patient derived ALL MLL-AF9 cells. MLL-AF9 ALL cells were treated with 0.3µM DSF and 1µM Cu for 16h. The proteins were detected using antibodies against the N-terminal MLL and vinculin. (**E**) Phase contrast photographs of patient derived ALL MLL-AF9 cells in a human mesenchymal stem cell co-culture with or without treatment. (**F**) Patient derived ALL MLL-AF9 cells were transduced to express the firefly luciferase and then seeded on MSCs for two weeks in presence of different doses of DS and 1µM of Cu. Bioluminescence was measured using the FLUOstar Omega microplate reader after adding the firefly luciferase substrate. Plot shows relative luminescence in treated and untreated cells.

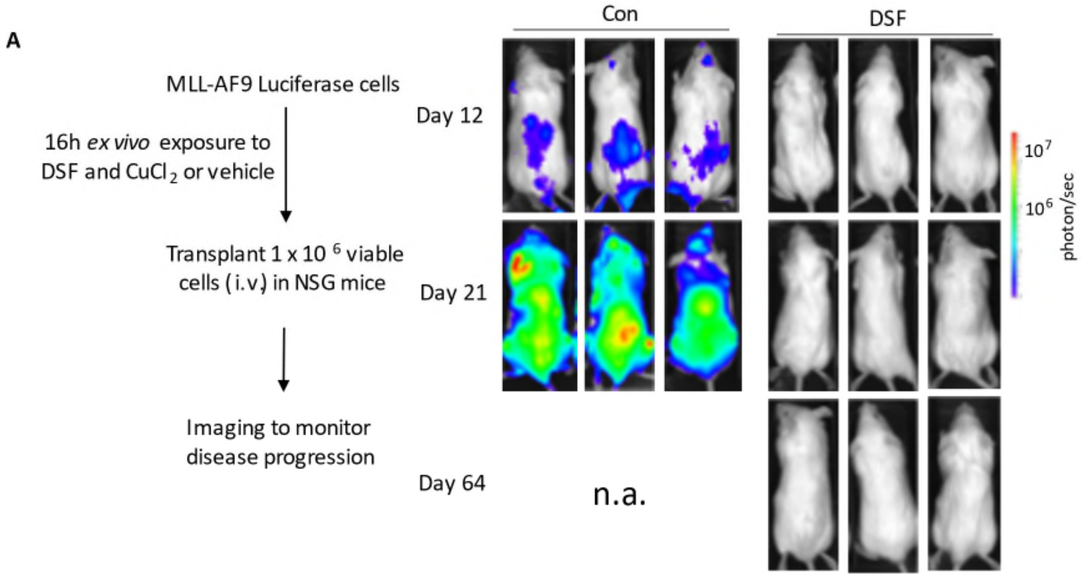

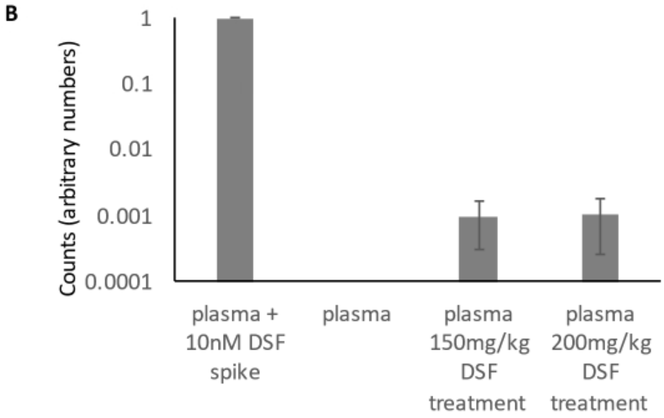

Fig. S3. Leukemic engraftment after ex vivo exposure to DSF-copper.

(A) Bioluminescence imaging of NSG recipient mice 10 days after injection with one million THP-1-Luciferase cells either treated with DSF/Cu or vehicle control for 16h. **(B)** Relative DSF concentration in plasma DSF or Con treated mice established with Liquid chromatography–mass spectrometry analysis.

Figure S4

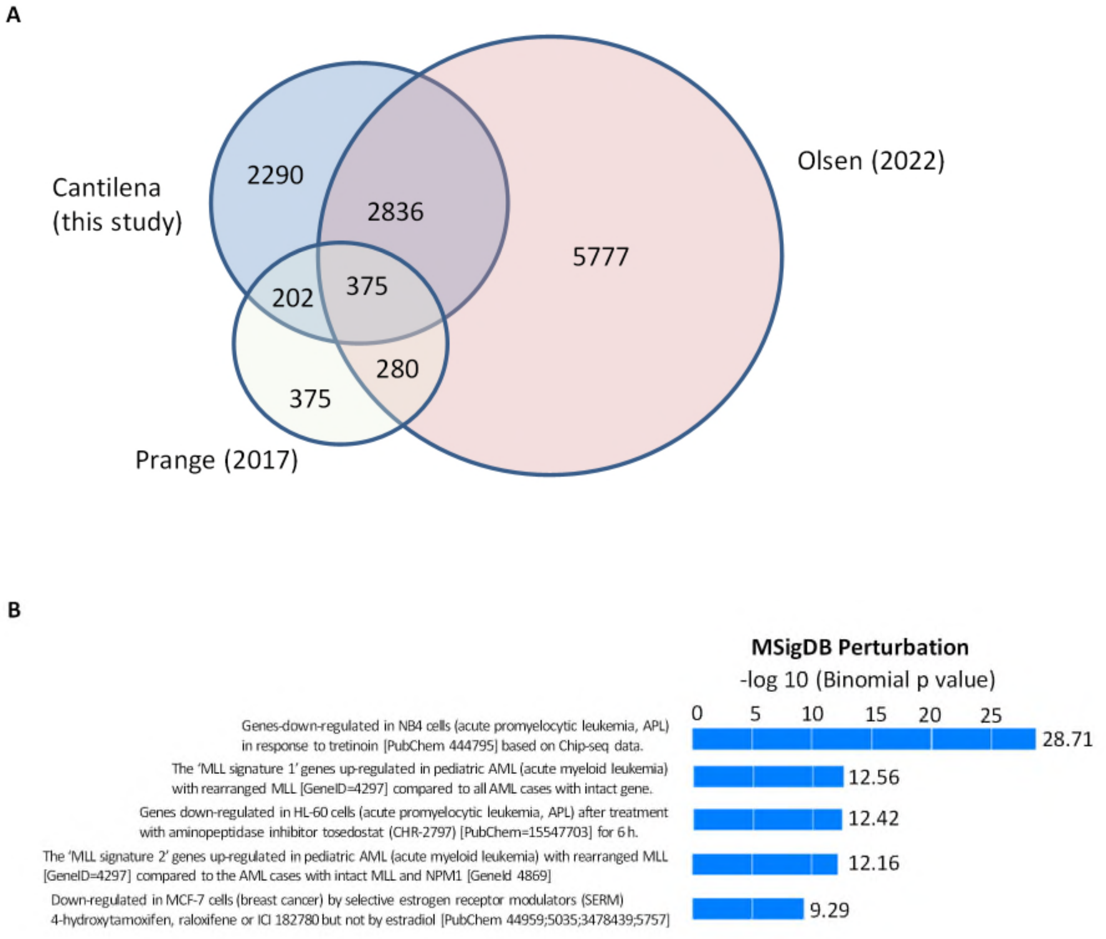

Fig. S4. Correlation of epigenetic changes with leukemic transcriptional programs.

(A) Venn diagram of the MLL-AF9 binding sites identified in this study and Prange *et al* (2017) and Olsen *et al* (2022). **(B)** The perturbation analysis showed enrichment of the 873 genes in APL and in *MLL* rearranged AML gene sets.
